# HOXA11 antisense long noncoding RNA (HOXA11‐AS): A promising lncRNA in human cancers

**DOI:** 10.1002/cam4.1571

**Published:** 2018-07-10

**Authors:** Cheng‐Wei Lu, Dan‐Dan Zhou, Tian Xie, Ji‐Long Hao, Om Prakash Pant, Cheng‐Bo Lu, Xiu‐Fen Liu

**Affiliations:** ^1^ Department of Ophthalmology The First Hospital of Jilin University Jilin China; ^2^ Department of Radiology The First Hospital of Jilin University Jilin China; ^3^ Department of Neurosurgery The People's Hospital of Jilin Province Jilin China; ^4^ Department of Cardiology The First Hospital of Jiamusi University Heilongjiang China

**Keywords:** HOXA11‐AS, long noncoding RNAs, tumor accelerator, tumor suppressor, tumorigenesis

## Abstract

The cancers are the leading cause of disease‐related deaths worldwide with a high risk of morbidity and mortality. Long noncoding RNAs (lncRNAs) play a critical role in a wide range of biological processes, including tumorigenesis. HOXA11‐AS (NCRNA00076), the antisense strands of HOXA11 gene, was initially revealed in a mouse embryonic cDNA library in 2009 and it was a fairly novel lncRNA. This review summarized the advanced research progression concerning the expression and role of HOXA11‐AS in different human malignancies. The expression of HOXA11‐AS is aberrantly altered in many cancers, either as a tumor suppressor or as a tumor accelerator. The different underlying mechanism of HOXA11‐AS in different cancers (including, nonsmall cell lung cancers, osteosarcoma, uveal melanoma, glioma, hepatocellular carcinoma, gastric cancer, breast cancer, cervical cancer, ovarian cancer, colorectal cancer, ovarian cancer, and glioblastoma) was also detailed. These findings lead us to conclude that HOXA11‐AS participate in the complex network of cancers and plays an important role in the tumorigenesis and progression. Functional HOXA11‐AS could be a promising biomarker for early detection as well as prognosis evaluation in cancer patients. Future HOXA11‐AS‐targeted intervention may become a valuable novel therapeutic tool, improving the clinical management of cancers.

## 1 INTRODUCTION

Cancer is a group of malignant diseases with uncontrolled cell growth, migration, and invasion. There are several types of cancers affecting the various organ of human body, such as lung cancer, gastric cancer (GC), and ovarian cancer. In 2015, an estimated 90.5 million people were diagnosed with cancer and have an incidence of 14.1 million new cases per year, 15.7% of all human death are caused by cancer^1^ Present therapeutic methods for advanced‐stage cancers still have some limitations and early‐stage cancers tend to have a poor diagnosis due to the lack of efficient biomarkers. Therefore, it is in urgent to explore available biomarkers as well as further investigate the molecular pathogenic mechanism regarding cancer invasion and metastasis, which may provide a vital theoretical basis for clinical cancer management.

In human, more than 70% genome is transcribed. Among these transcripts, only 2% are protein‐coding genes and the rest are noncoding RNAs (ncRNAs). Unlike short ncRNAs (rRNA, tRNA, snoRNA, snRNA, miRNAs), lncRNA is considered as a class of ncRNAs with more than 200 nucleotides. LncRNA were initially underestimated for a long time as a transcriptional noise in the genome. Recently, emerging evidence demonstrates that lncRNAs play a critical role in a wide range of biological processes, such as cell proliferation, differentiation, migration, invasion, apoptosis, reprogramming of stem cell pluripotency and etc. The expression patterns of many lncRNAs are altered in diverse human malignancies, and they may serve either as a tumor suppressor or as a tumor accelerator depending upon the type of cancer or the environment. lncRNAs are emerging as new biomarkers in many human malignancies. Revealing the complex mechanisms of lncRNAs in tumorigenesis would facilitate the prevention, prognostic evaluation, and treatment of cancers. Until this time, only a small portion of lncRNAs have been well elucidated, and the majority of the lncRNAs remain largely unknown, which need to be further investigated.

Homeobox (HOX) gene family is characterized by highly conserved homeodomains which enable HOX proteins to bind to specific DNA regions and regulate other genes’ transcription during tumorigenesis and embryogenesis. Dysregulated HOX gene expressions were found in various human cancers, such as lung cancer,^2^ bladder cancer.^3^

In human HOX gene family, there are four clusters (A, B, C, and D) located on four different chromosomes (7p15, 17p21, 12q13, and 2q31, respectively).^4^, ^5^ There are 39 HOX genes in HOX gene family, and these genes are assigned to 13 paralog groups in each cluster, based on the position within the cluster and the homeobox sequence similarity. Each cluster has 9‐11 genes.

There are three lncRNAs (HOXA11‐AS, HOXA10‐AS, and HOXA transcript at the distal tip (HOTTIP) at the 5′ region of the HOXA cluster. Among these three, HOXA‐11AS and HOTTIP are frequently studied, while HOXA 10‐AS is relatively rarely studied. HOTTIP expression was significantly increased in skin, hepatocellular, pancreatic, lung and tongue squamous cell cancer tissues. Overexpression of HOTTIP was positive correlated with tumor stage, poor overall survival, distant metastasis and lymph node metastasis, indicating that HOTTIP expression may serve as a potentical biomarker for poor prognosis in cancers.^6^

Besides HOTTIP, HOX transcript antisense RNA (HOTAIR) is also most frequently studied HOX family member in this area. HOTAIR is a lncRNA which is expressed in the HOXC locus and has been reported to be a pro‐oncogenic factor and a negative prognostic factor in several types of cancer, including breast, pancreatic, gastric, colorectal and bladder cancer. Above studies indicate that lncRNAs from HOX family may play important roles in cancers, but the expression pattern and roles may vary in different types of cancers.

Growing researches demonstrate that aberrantly expressed HOXA11‐AS play key roles in the development and progression of cancers. In this review, we focused on the researches progresses and advances of HOXA11‐AS in cancers due to its potential roles in human malignancies. HOXA11‐AS, also known as NCRNA00076, is initially discovered in a mouse embryonic cDNA library using a probe from the sense HOXA11 cDNA sequences.^7^ HOXA11‐AS might function by transcriptional regulation of HOXA11 expression in cervix cancer.^8^ Although the specific underlying mechanisms are not elucidated yet, the signaling pathways by which HOXA11‐AS is involved in cancers may be explored as a future therapeutic approach for the management of human malignancies.

## 2 HOXA11‐AS LNCRNA IN HUMAN CANCERS

### 2.1 Nonsmall cell lung cancers

Lung carcinomas which compromise small cell lung cancers (SCLCs), as well as Nonsmall cell lung cancers (NSCLCs) are highly aggressive malignant cancers, and hazardous to human health. Nonsmall cell lung cancers are the most common variety, accounting for 80%‐85% of new LC cases. Approximately, 70% of NSCLCs patients are diagnosed at an advanced stage with the tumor already being metastasized and its 5‐year survival rate is about 16%.^9^ Therefore, it is urgent to explore the exact molecular processes involved in the development of NSCLCs and to achieve potential therapeutic targets.

HOXA11‐AS was notably highly expressed in squamous cell carcinoma and lung adenocarcinoma. HOXA11‐AS may play an important role in development as well as the progression of NSCLCs through regulation of numerous pathways and genes. Zhang et al revealed that HOXA11‐AS was markedly overexpressed in NSCLC tissues as well as cells both in vivo and vitro. Moreover, knockdown of HOXA11‐AS inhibited the proliferation, migration, invasion, tumorigenic as well as the angiogenic capability of NSCLC cells and induced apoptosis. HOXA11‐AS also leads to cell cycle arrest at G0/G1 or G2/M phase.^10^ In another study, Chen et al concluded that HOXA11‐AS was considerably upregulated in NSCLC tissues, compared with that of normal tissues. Moreover, higher expression of HOXA11‐AS was correlated with TMN staging as well as lymph node spread, resulting in poor prognosis. They further stated that HOXA11‐AS was also engaged in the NSCLC cell invasion along with epithelial‐mesenchymal transition (EMT) process and knockdown of HOXA11‐AS in NSCCL cells inhibited cell invasive ability combined with decreased the expression of EMT‐related transcription factors by means of repressing miR‐200b through interacting with zeste homolog 2 (EZH2) and DNMT1 in NSCLC.^11^ Additionally, HOXA11‐AS might play an important role in NSCLC development as well as progression through regulating the expression of numerous pathways and genes, especially DOCK8 and TGF‐beta (TGF‐β) pathway.^12^ As a conclusion, these results indicate that HOXA11‐AS plays a key role in NSCLC and it can be a novel therapeutic target for the treatment of NSCLC. However, further functional experiments are needed to verify the exact molecular mechanism involved in the role of HOXA11‐AS in NSCLC carcinogenesis as well as its progression.

### 2.2 Osteosarcoma

Osteosarcoma (OS) is an aggressive malignant neoplasm that arises from the primitive bone‐forming mesenchymal cells. It is the most common type of primary bone malignancy and is mainly prevalent in the pediatric age group. Even though there is a current advancement in multimodal treatment options, such as tumor excision as well as adjuvant chemotherapy, survival expectancy is still low. The underlying process of the formation along with the progression of OS is vague. Thus, further identifying the biomarker of OS progression is vital for improving the diagnosis along with the treatment of OS.

Cui et al stated that the overexpression of HOXA11‐AS was noted to be upregulated both in OS tissues and cell lines (KHOS MG‐63 and U2OS). Higher expression of HOXA11‐AS was revealed to be associated with advanced clinical stage, distant metastasis as well as the poor survival of OS patients. HOXA11‐AS silence suppressed cell proliferation as well as invasion and induced cell cycle arrest in the G0/G1 phase in OS cells. Furthermore, HOXA11‐AS was revealed to bind with miR‐124‐3p directly and increase ROCK1 expression, leading to the proliferation and invasion of OS cells. However, the expression, as well as the underlying mechanism of miR‐124‐3p in OS, is still unclear, and further investigation is required. These findings indicate that HOXA11‐AS may serve as a tumor accelerator via promoting cell growth and invasion in OS progression. Still, further investigation into HOXA11‐AS‐ miR‐124‐3p‐ROCK1 signaling pathway may facilitate the development of the new therapeutic strategies for OS treatment.^13^

### 2.3 Uveal melanoma

Melanoma is a type of malignant cancer that originates from the melanocytes, which may occur in the human skin, eye, intestines, mouth and etc. Uvea is the second most common site for melanoma following the skin, and Uveal melanoma (UM) is known as the most common type of primary intraocular tumor in adults. UM possesses high metastatic ability that may cause up to 50% patients liver metastasis.^14^ In‐depth understanding of the melanomagenesis, both epigenetic and genetic, may assist in the prevention, diagnosis, and intervention for UM.

LncRNAs can alter gene regulation and lead to epigenetic changes. HOXA11‐AS was found to be overexpressed in UM tissues as well as cell lines. HOXA11‐AS promotes UM cell proliferation along with invasion and it inhibits apoptosis of UM cell. LncRNAs (eg PVT1) may regulate gene expression via the interactions with enhancer of EZH2.^15^ HOXA11‐AS was also found to be directly bound to EZH2 by RIP assay. Knockdown of HOXA11‐AS increased cell cycle regulation gene p21 (target of EZH2) expression, and knockdown of EZH2 upregulated p21 expression levels in UM cells. ChIP assays revealed that HOXA11‐AS recruited EZH2 to the p21 promoter region and suppressed its transcription by regulating H3K27me3. These results suggest that lncRNA HOXA11‐AS is involved in EZH2‐mediated repression of p21 in UM cells. MiR‐124 was detected to be significantly decreased in UM tissues, and overexpression of miR‐124 impaired UM cell proliferation as well as invasion. HOXA11‐AS was also found to function as a competing endogenous RNA and sponged miR‐124 in UM cells. Additionally, miR‐124 reversed the promoting effect of HOXA11‐AS on proliferation and invasion of UM cells. These indicate that HOXA11‐AS may serve an oncogenic biomarker for UM.

### 2.4 Glioma

Glioma is a malignant tumor that arises from the glial cells. Gliomas account up to 30% of all brain and central nervous system (CNS) malignancies and an estimated 80% of all malignant brain tumors.^16^

HOXA11‐AS was significantly overexpressed in surgically excised glioma tissue compared with adjacent nontumor tissue.^17^, ^18^ And elevated HOXA11‐AS indicates a short survival and a poor prognosis in glioma patients.^17^, ^18^ The significant difference in HOXA11‐AS expression among glioblastoma (GBM) subtypes revealed by the mRNA microarray may indicate that HOXA11‐AS may also serve as a marker for glioma molecular subtype.^17^ The expression level of HOXA11‐AS was also significantly elevated in glioma cell lines (U251 and SHG44) when compared with normal human astrocytes. By employing the siRNA transfection, downregulation of the HOXA11‐AS expression repressed the cell proliferation via inducing cell cycle arrest at the G0/G1 phase and promoted the apoptosis in glioma cell lines as well as xenograft mouse model.^17^, ^18^ MiR‐140‐5p was downregulated in glioma cells, and miR‐140‐5p was found to be directly targeted HOXA11‐AS at 3′‐UTR via the bioinformatics analysis and luciferase assay. Furthermore, miR‐140‐5p inhibitor rescued the effect of HOXA11‐AS on proliferation and apoptosis. Thus, these results indicate that HOXA11‐AS may serve as a prognostic evaluation biomarker for glioma patients, and HOXA11‐AS sponging miR‐140‐5p might play a vital role in the pathogenesis of glioma.

### 2.5 Hepatocellular carcinoma

Hepatocellular carcinoma (HCC) is one of the most common primary carcinomas of the liver. HOXA11‐AS was comparatively highly expressed in HCC tissues as well as cells detected by qRT‐PCR assays.^19^ Yu et al found that HOXA11‐AS could hinder HCC cell proliferation along with the cell cycle progression from G1 to G0 phase, and induce their apoptosis. LATS1 genes were also detected as the downstream target genes for HOXA11‐AS, which could be inhibited by HOXA11‐AS via linking EZH2 proteins enhancers.^19^

In conclusion, HOXA11‐AS may act as an oncogene in HCC development. Interactions between HOXA11‐AS and LATS1 may provide a new prognostic marker and a therapeutic target for HCC.

### 2.6 Gastric cancer

Among the malignant cancers, GC is ranked as the most common cancer worldwide. GC has high mortality and is the second most common cause of cancer‐related death worldwide.^20^ The epidemiology study demonstrated that the environmental factors and lifestyles are vital etiology factors of GC.^21^ Numerous genetic modifications contribute to the onset of GC, including oncogenes, tumor suppressor genes, as well as growth factors.^22^

Overexpressed HOXA11‐AS was detected in human GC tissues when compared to matched normal tissues.^23^ Liu et al found that knockdown of HOXA11‐AS hindered GC cell proliferation along with the cell cycle progression from G1 to G0 phase, and suppressed GC cells migration as well as invasion in vivo. Besides, the mechanistic investigation revealed that HOXA11‐AS could have an interaction with WDR5 and stimulate the transcription of β‐catenin as well as binds with EZH2 and inhibits the transcription of P21. Additionally, HOXA11‐AS induces the degradation of KLF2 mRNA through interacting with STAU1.^23^

Sun et al reported that patients with high HOXA11‐AS expression had a shorter survival and poorer prognosis.^24^ HOXA11‐AS alterations showed a complexly integrated phenotype affecting cell growth, migration, invasion, and apoptosis both in vitro as well as in vivo. Systematically, these findings support a model in which the EZH2/HOXA11‐AS/LSD1 complex and HOXA11‐AS/miR‐1297/EZH2 cross‐talk aid as key effectors in GC tumorigenesis as well as progression. This finding may signify novel therapeutic directions in GC.^24^

In conclusion, HOXA11‐AS has found to be linked with tumor suppressor or oncogenic pathways of GC, whereas altered expression of HOXA11‐AS was linked with the incidence as well as the development of GC. This finding presents evidence that HOXA11‐AS may be considered as a candidate detection biomarker as well as a novel therapeutic target in GC.

### 2.7 Breast cancer

Breast cancer is one of the most common cancers and is prevalent in females.^25^ Although chemotherapy, operative treatment, along with molecular targeting treatments are improved, the prognosis of breast cancer is not satisfactory.^26^

Li et al reported that the expression of HOXA11‐AS in breast cancer tissue was higher than that in tissue adjacent to cancer.^27^ Interfering in HOXA11‐AS could stimulate the apoptosis of breast tumor cell as well as inhibit the invasion and migration capacity of tumor cell through affecting EMT‐related molecular markers expressions such as E‐cadherin, N‐cadherin, Vimentin.^27^

In conclusion, HOXA11‐AS may be explored as a vital molecular target for preventing the metastasis of breast cancer in clinical practice. HOXA11‐AS may be a promising tumor biomarker for early detection, and a potential therapeutic target for breast cancer patients.

### 2.8 Cervical cancer

Cervical cancer is a malignant tumor arising from the cervix. HOXA11‐AS was first discovered to be abnormally expressed in human uterine cervical cancer by Jie Chen's team in 2015^28^ via systematic gene microarray analysis, along with many other lncRNAs. Kim et al in 2016 further elucidated the expression as well as the underlying mechanism of HOXA11‐AS in cervical cancer. HOXA11‐AS expression was dramatically higher in human cervical cancer tissues than corresponding normal controls. HOXA11‐AS was proved to be an independent prognosticator of cervical cancer patients, and higher expression of HOXA11‐AS correlates with poor survival. HOXA11‐AS expression varied in different subtypes of cervical cancer cells, for example,it was higher in epidermoid cervical carcinoma established from a metastasis in the small bowel mesentery (CaSki), squamous cervical carcinoma (SiHa) cells, and epitheloid cervical carcinoma (HeLa), than that in HPV negative cervical carcinoma (C33A) cells and epidermoid cervical carcinoma (ME‐180). Knockdown of HOXA11‐AS with siRNA interference suppressed cell proliferation in cervical cancer cells (HeLa and CaSki) cultured in vitro and xenograft tumor growth in mice, illustrating that HOXA11‐AS is involved in the pathogenesis of cervical cancer. HOXA11‐AS overexpression/know down studies further demonstrated that high expression of HOXA11‐AS induced cell migration and invasion in cervical cancer cells (HeLa) via the upregulation of vascular endothelial growth factor (VEGF), matrix metalloproteinase 2 (MMP‐2), matrix metalloproteinase 9 (MMP‐9), and the modification of EMT‐related genes (decreased E‐cadherin, increased β‐catenin and increased vimentin expression). Thus, HOXA11‐AS may be potential biomarkers for prognostic evaluation. Further studies orientated with VEGF, MMPs, and EMT pathways may facilitate the explorement of the therapeutic target for cervical cancer treatment.

### 2.9 Colorectal cancer

Colorectal cancer (CRC) is the third most common malignancy and has the third highest mortality rate in the United States.^29^ Although the death rate has decreased for decades owing to the implementation of screening strategies along with the improvement of standard treatment,^30^ the occurrence of relapses as well as the unfavorable prognosis still influences the consequence of treating CRC.

Li et al found that the expression of HOXA11‐AS in CRC tissues and cell lines was decreased in comparison with that of the controls. Clinicopathological analysis showed that lower expression of HOXA11‐AS was remarkably linked with tumor size, advanced tumor‐node‐metastasis stage, lymphnode metastasis as well as carcinoembryonic antigen level of patients with CRC. Nevertheless, this was based on a narrow number of patients and future studies investigating more patient samples are required.^31^

In conclusion, HOXA11‐AS may act as a vital diagnostic marker as well as the therapeutic target of CRC, and further analyses are needed to confirm these findings.

### 2.10 Glioblastoma

Among the primary brain cancers GBM, a grade IV glioma, is the most common, aggressive and lethal cancer. The only treatment option for GBM is aggressive surgical resections because it is resistant to radiotherapy as well as chemotherapy. Regardless of latest advances in treating GBM, treatments for GBM remain palliative and do slight to modify the poor prognosis due to this cancer.^32^ The prognosis for GBM is very poor, with a mean survival of 12‐18 months. Hence, it is urgent to find effective biomarkers, which will contribute a key role in the diagnosis and treatment of GBM.

The down‐regulation of HOXA11 is linked with a poor prognosis in GBM patients. The aberrant expression of HOXA11 has been related to the prognosis of numerous cancers, comprising GBM. Ben se et al revealed the tumor suppressor role of HOXA11 in GBM patients on both in vitro experiments and human tissues. They also suggested that in GBM the epigenetic down‐regulation ratio of HOXA11 was 51%‐75%, in addition, HOXA11 was one of the commonly methylated genes in GBM. The methylation of HOXA11 was related to the older patient as well as poor survival in GBM. Furthermore, this study discovered candidate mediators (TGFBR2, CRIM1, TXNIP, DPYSL2, and CRMP1) that may impart treatment resistance following HOXA11 suppression.^33^ As a conclusion, these results indicate that HOXA11‐AS plays a key role in GBM and it can be a novel therapeutic target for the treatment of GBM. Although additional study will be desired to endorse the value of HOXA11 as a possible target for overwhelming the treatment resistance by developing chemo‐ or radiosensitizers.

### 2.11 Ovarian cancer

Ovarian cancer is a malignant tumor that arises in or on an ovary. Arising in the cell lining of the ovary, epithelial ovarian cancer is the most common type of ovarian cancer. The HOXA region of protein‐coding genes impacts ovarian carcinogenesis. Richards et al in 2015 reported that HOXA11‐AS expression levels were significantly decreased in human epithelial ovarian cancer than normal ovarian tissues, indicating that HOXA11‐AS may serve as a tumor suppressor in epithelial ovarian cancer.^4^ Instead of a tumor suppressor, HOXA11‐AS was served as a tumor accelerator in serous ovarian cancer (SOC), which is the most common histological form of epithelial ovarian cancer.^34^ HOXA11‐AS expression in SOC tissue was found to be dramatically higher (77‐fold) than that of normal ovarian tissues by qRT‐PCR. In addition, higher HOXA11‐AS expression was significantly associated with higher histological grade, higher cancer antigen 125 (CA125) level and poor patient survival.^34^ HOXA11‐AS was proved by siRNA transfection to promote cell proliferation, invasion, and migration in SOC cells (OVCAR3, OVCA429, and SKOV3) cultured in vitro.^34^ Moreover, HOXA11‐AS was verified to induce SOC cell migration and invasion through the upregulation of MMP‐2, MMP‐9, and VEGF.^34^ Epithelial marker (E‐cadherin) was significantly upregulated, and mesenchymal marker (N‐cadherin, β‐catenin, and vimentin) as well as the expression of the EMT modulator (Twist and Snail) were downregulated in siHOXA11‐AS‐transfected cells,^34^ indicating that HOXA11‐AS may serve as a prognostic factor for SOC patients and indicate its potential in promoting tumor progression by regulating the VEGF, MMPs, and EMT‐relevant mechanisms. In conclusion, the expression and role of HOXA11‐AS vary in different studies, it may due to the different subtypes of ovarian cancer, and more in‐depth researches are needed.

## 3 CONCLUSIONS AND FUTURE PERSPECTIVES

**Figure 1:**
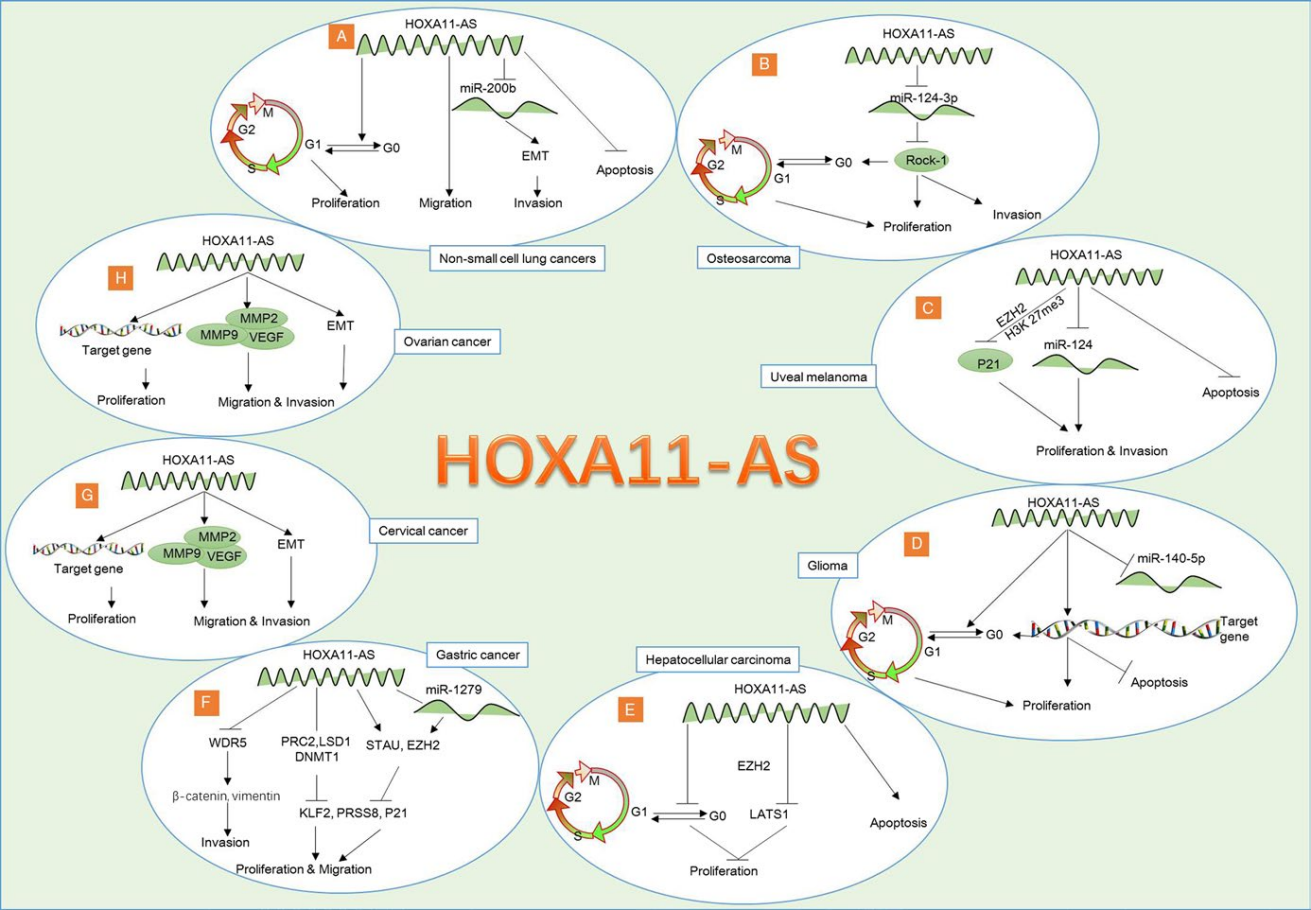
The different regulatory mechanisms of HOXA11‐AS in different human cancers. A, Nonsmall cell lung cancers: HOXA11‐AS promotes cell proliferation, migration, invasion and inhibit apoptosis. HOXA11‐AS promoted EMT by inhibiting miR‐200b expression. B, Osteosarcoma: HOXA11‐AS binds with miR‐124‐3p directly and increased ROCK1 expression, leading to the cell proliferation and invasion. C, Uveal melanoma: HOXA11‐AS interacts with enhancer of EZH2 to suppress its target p21 protein expression. It also promotes proliferation and invasion by inhibiting miR‐124. D, Glioma: HOXA11‐AS increased the cell proliferation via promoting cell cycle and inhibited the apoptosis. miR‐140‐5p was downregulated in glioma cells and directly targets HOXA11‐AS at 3′‐UTR. miR‐140‐5p inhibitor rescued the effect of HOXA11‐AS on proliferation and apoptosis. E, Hepatocellular carcinoma (HCC): HOXA11‐AS inhibit the proliferation of HCC cells via the retardation of the cell cycle progression from G1 to G0 phase, and promote their apoptosis. HOXA11‐AS inhibits the expression of LATS1 genes by binding enhancer of EZH2 protein. F, Gastric cancer: HOXA11‐AS binds RNA‐binding proteins (PRC2, LSD1, and DNMT1), and inhibited PRSS8 and KLF2 expression, leading to the increased proliferation and migration in gastric cancer cells. HOXA11‐AS repressed KLF2 and P21 expression by interacting with STAU1 and EZH2, leading to the increased cell proliferation and invasion. HOXA11‐AS binds with miR‐1297, resulting in elevated EZH2 expression. HOXA11‐AS further recruits EZH2 to repress PRSS8 and KLF2 transcription.HOXA11‐AS binds with WDR5 and promotes β‐catenin, vimentin expressions, leading to increased invasion ability. G, Cervical cancer: HOXA11‐AS promotes cell proliferation. HOXA11‐AS induced cell migration and invasion via the upregulation of VEGF, MMP‐2, MMP‐9, and the modification of EMT‐related genes (decreased E‐cadherin, increased β‐catenin and increased vimentin expression). H, Ovarian cancer: HOXA11‐AS promotes cell proliferation. HOXA11‐AS induced cell migration and invasion via the upregulation of VEGF, MMP‐2, MMP‐9, and the modification of EMT‐related genes (decreased E‐cadherin, and increased N‐cadherin, β‐catenin, vimentin, Twist and Snail expression)

Dysregulation of ncRNAs is involved in malignant cells, leading to cancer progressive, indicating that ncRNAs may be a new answer for cancers. The roles of lncRNAs in human malignancies have long been a subject of interest to various authors. HOXA11‐AS is a lncRNA located in the HOXA gene cluster and the clinical significance, as well as its’ molecular mechanisms in controlling cancers, are unclear. Overview of HOXA11‐AS in human tumors may provide new insights into the mechanisms of cancer development. This review elucidates the advanced researches and progresses with the possible role of HOXA11‐AS in different human cancers (Table 1), as well as the underlying involved mechanisms (Figure 1). Studies showed that the expression pattern and role of HOXA11‐AS varies in different types of cancers, either as an oncogene or tumor suppressor. The tissue and mechanistic heterogeneity of HOXA‐11AS in different cancers is just beginning to emerge. One of the important future challenges is to solve this puzzle. Highly expressed HOXA11‐AS correlates with poor survival in some cancers patients, such as glioma, OS, cervical cancer, epithelial ovarian cancer, and glioblastoma, indicating that HOXA11‐AS may serve as a biomarker for early detection and prognosis evaluation. HOXA11‐AS is involved in proliferation, migration, invasion, apoptosis and epithelial to the mesenchymal transition process. HOXA11‐AS‐oriented interventions may enhance the clinical management of cancers, though further studies are needed.

**Table 1:** Functional characterization of HOXA11‐AS in various tumors

Tumor type	Expression	Role	Related molecules	Phenotypes affected	References
Nonsmall cell lung cancers	Upregulation	Tumor accelerator	MiR‐200b, EZH2, DNMT1	Proliferation, migration, invasion, apoptosis, EMT	10, 11
Osteosarcoma			MiR‐124‐3p, ROCK1	Proliferation, invasion	13
Uveal melanoma			P21, miR‐124, H3K27me3	Proliferation, invasion, apoptosis	35
Glioma			MiR‐140‐5p	Proliferation, cell cycle, apoptosis	17, 18
Hepatocellular carcinoma			EZH2,LATS1, H3K27	Proliferation, apoptosis	19
Gastric cancer			PRC2, LSD1, DNMT1, EZH2/HOXA11‐AS/LSD1, HOXA11‐AS/miR‐1297/EZH2 cross‐talk	Proliferation, migration, invasion, and apoptosis	23, 24
Breast cancer			E‐cadherin, N‐cadherin, Vimentin	Proliferation, invasion	27
Cervical cancer			MMP‐2, MMP‐9, VEGF, E‐cadherin, β‐catenin, vimentin	Proliferation, migration, invasion and EMT	28, 36
Colorectal cancer	Downregulation	Tumor accelerator			31
Glioblastoma		Tumor suppressor	TGFBR2, CRIM1, TXNIP, DPYSL2, CRMP1	Migration and invasion	33
Ovarian cancer	Upregulation	Tumor accelerator	MMP‐2, MMP‐9, VEGF, β‐catenin, E‐cadherin, Snail, Twist, vimentin	Proliferation, migration, invasion and EMT	34
	Downregulation	Tumor suppressor	T allele expression and variant	Proliferation, migration and invasion	4

## CONFLICT OF INTEREST

Authors declare no conflicts of interest for this article.

## References

[1] Mortality GBD , Causes of Death C . Global, regional, and national life expectancy, all‐cause mortality, and cause‐specific mortality for 249 causes of death, 1980‐2015: a systematic analysis for the Global Burden of Disease Study 2015. Lancet. 2016;388:1459‐1544. 27733281 10.1016/S0140-6736(16)31012-1 PMC5388903

[2] Calvo R , West J , Franklin W , et al. Altered HOX and WNT7A expression in human lung cancer. Proc Natl Acad Sci USA. 2000;97:12776‐12781. 11070089 10.1073/pnas.97.23.12776 PMC18840

[3] Cantile M , Cindolo L , Napodano G , Altieri V , Cillo C . Hyperexpression of locus C genes in the HOX network is strongly associated in vivo with human bladder transitional cell carcinomas. Oncogene. 2003;22:6462‐6468. 14508527 10.1038/sj.onc.1206808

[4] Richards EJ , Permuth‐Wey J , Li Y , et al. A functional variant in HOXA11‐AS, a novel long non‐coding RNA, inhibits the oncogenic phenotype of epithelial ovarian cancer. Oncotarget. 2015;6:34745‐34757. 26430965 10.18632/oncotarget.5784 PMC4741487

[5] Grier DG , Thompson A , Kwasniewska A , McGonigle GJ , Halliday HL , Lappin TR . The pathophysiology of HOX genes and their role in cancer. J Pathol. 2005;205:154‐171. 15643670 10.1002/path.1710

[6] Fan Y , Yan T , Chai Y , Jiang Y , Zhu X . Long noncoding RNA HOTTIP as an independent prognostic marker in cancer. Clin Chim Acta. 2017; S0009‐8981(17)30292‐9. 10.1016/j.cca.2017.07.031. [Epub ahead of print] 28778381

[7] Chau YM , Pando S , Taylor HS . HOXA11 silencing and endogenous HOXA11 antisense ribonucleic acid in the uterine endometrium. J Clin Endocrinol Metab. 2002;87:2674‐2680. 12050232 10.1210/jcem.87.6.8527

[8] Dear TN , Sanchez‐Garcia I , Rabbitts TH . The HOX11 gene encodes a DNA‐binding nuclear transcription factor belonging to a distinct family of homeobox genes. Proc Natl Acad Sci USA. 1993;90:4431‐4435. 8099440 10.1073/pnas.90.10.4431 PMC46525

[9] Chen G , Umelo IA , Lv S , et al. miR‐146a inhibits cell growth, cell migration and induces apoptosis in non‐small cell lung cancer cells. PLoS ONE. 2013;8:e60317. 23555954 10.1371/journal.pone.0060317 PMC3608584

[10] Zhang Y , Chen WJ , Gan TQ , et al. Clinical significance and effect of lncRNA HOXA11‐AS in NSCLC: a study based on bioinformatics, in vitro and in vivo verification. Sci Rep. 2017;7:5567. 28717185 10.1038/s41598-017-05856-2 PMC5514100

[11] Chen JH , Zhou LY , Xu S , Zheng YL , Wan YF , Hu CP . Overexpression of lncRNA HOXA11‐AS promotes cell epithelial‐mesenchymal transition by repressing miR‐200b in non‐small cell lung cancer. Cancer Cell Int. 2017;17:64. 28615992 10.1186/s12935-017-0433-7 PMC5468943

[12] Zhang Y , He RQ , Dang YW , et al. Comprehensive analysis of the long noncoding RNA HOXA11‐AS gene interaction regulatory network in NSCLC cells. Cancer Cell Int. 2016;16:89. 27980454 10.1186/s12935-016-0366-6 PMC5133743

[13] Cui M , Wang J , Li Q , Zhang J , Jia J , Zhan X . Long non‐coding RNA HOXA11‐AS functions as a competing endogenous RNA to regulate ROCK1 expression by sponging miR‐124‐3p in osteosarcoma. Biomed Pharmacother. 2017;92:437‐444. 28558357 10.1016/j.biopha.2017.05.081

[14] Coupland SE , Lake SL , Zeschnigk M , Damato BE . Molecular pathology of uveal melanoma. Eye. 2013;27:230‐242. 23222563 10.1038/eye.2012.255 PMC3574255

[15] Kong R , Zhang EB , Yin DD , et al. Long noncoding RNA PVT1 indicates a poor prognosis of gastric cancer and promotes cell proliferation through epigenetically regulating p15 and p16. Mol Cancer. 2015;14:82. 25890171 10.1186/s12943-015-0355-8 PMC4399399

[16] Goodenberger ML , Jenkins RB . Genetics of adult glioma. Cancer Genet. 2012;205:613‐621. 23238284 10.1016/j.cancergen.2012.10.009

[17] Wang Q , Zhang J , Liu Y , et al. A novel cell cycle‐associated lncRNA, HOXA11‐AS, is transcribed from the 5‐prime end of the HOXA transcript and is a biomarker of progression in glioma. Cancer Lett. 2016;373:251‐259. 26828136 10.1016/j.canlet.2016.01.039

[18] Cui Y , Yi L , Zhao JZ , Jiang YG . Long noncoding RNA HOXA11‐AS functions as miRNA sponge to promote the glioma tumorigenesis through targeting miR‐140‐5p. DNA Cell Biol. 2017;36:822‐828. 28832185 10.1089/dna.2017.3805

[19] Yu J , Hong JF , Kang J , Liao LH , Li CD . Promotion of LncRNA HOXA11‐AS on the proliferation of hepatocellular carcinoma by regulating the expression of LATS1. Eur Rev Med Pharmacol Sci. 2017;21:3402‐3411. 28829501

[20] Torre LA , Bray F , Siegel RL , Ferlay J , Lortet‐Tieulent J , Jemal A . Global cancer statistics, 2012. CA Cancer J Clin. 2015;65:87‐108. 25651787 10.3322/caac.21262

[21] Crew KD , Neugut AI . Epidemiology of gastric cancer. World J Gastroenterol. 2006;12:354‐362. 16489633 10.3748/wjg.v12.i3.354 PMC4066052

[22] Jemal A , Bray F , Center MM , Ferlay J , Ward E , Forman D . Global cancer statistics. CA Cancer J Clin. 2011;61:69‐90. 21296855 10.3322/caac.20107

[23] Liu Z , Chen Z , Fan R , et al. Over‐expressed long noncoding RNA HOXA11‐AS promotes cell cycle progression and metastasis in gastric cancer. Mol Cancer. 2017;16:82. 28441948 10.1186/s12943-017-0651-6 PMC5405470

[24] Sun M , Nie F , Wang Y , et al. LncRNA HOXA11‐as promotes proliferation and invasion of gastric cancer by scaffolding the chromatin modification factors PRC2, LSD1, and DNMT1. Cancer Res. 2016;76:6299‐6310. 27651312 10.1158/0008-5472.CAN-16-0356

[25] Zabkiewicz C , Resaul J , Hargest R , Jiang WG , Ye L . Bone morphogenetic proteins, breast cancer, and bone metastases: striking the right balance. Endocr Relat Cancer. 2017;24:R349‐R366. 28733469 10.1530/ERC-17-0139 PMC5574206

[26] Gemignani ML , Hetzel DJ . Current advances in endocrine therapy options for premenopausal women with hormone receptor positive breast cancer. Gynecol Oncol. 2017;147:153‐157. 28662774 10.1016/j.ygyno.2017.06.023 PMC5639942

[27] Li W , Jia G , Qu Y , Du Q , Liu B , Liu B . Long non‐coding RNA (LncRNA) HOXA11‐AS promotes breast cancer invasion and metastasis by regulating epithelial‐mesenchymal transition. Med Sci Monit. 2017;23:3393‐3403. 28701685 10.12659/MSM.904892 PMC5521048

[28] Chen J , Fu Z , Ji C , et al. Systematic gene microarray analysis of the lncRNA expression profiles in human uterine cervix carcinoma. Biomed Pharmacother. 2015;72:83‐90. 26054679 10.1016/j.biopha.2015.04.010

[29] Siegel R , Desantis C , Jemal A . Colorectal cancer statistics, 2014. CA Cancer J Clin. 2014;64:104‐117. 24639052 10.3322/caac.21220

[30] Altobelli E , Lattanzi A , Paduano R , Varassi G , di Orio F . Colorectal cancer prevention in Europe: burden of disease and status of screening programs. Prev Med. 2014;62:132‐141. 24530610 10.1016/j.ypmed.2014.02.010

[31] Li T , Xu C , Cai B , Zhang M , Gao F , Gan J . Expression and clinicopathological significance of the lncRNA HOXA11‐AS in colorectal cancer. Oncol Lett. 2016;12:4155‐4160. 27895785 10.3892/ol.2016.5129 PMC5104251

[32] Seymour T , Nowak A , Kakulas F . Targeting aggressive cancer stem cells in glioblastoma. Front Oncol. 2015;5:159. 26258069 10.3389/fonc.2015.00159 PMC4507454

[33] Se YB , Kim SH , Kim JY , et al. Underexpression of HOXA11 is associated with treatment resistance and poor prognosis in glioblastoma. Cancer Res Treat. 2017;49:387‐398. 27456940 10.4143/crt.2016.106 PMC5398402

[34] Yim GW , Kim HJ , Kim LK , et al. Long non‐coding RNA HOXA11 antisense promotes cell proliferation and invasion and predicts patient prognosis in serous ovarian cancer. Cancer Res Treat. 2017;49:656‐668. 27737536 10.4143/crt.2016.263 PMC5512379

[35] Lu Q , Zhao N , Zha G , Wang H , Tong Q , Xin S . LncRNA HOXA11‐AS exerts oncogenic functions by repressing p21 and miR‐124 in Uveal Melanoma. DNA Cell Biol. 2017;36:837‐844. 28749709 10.1089/dna.2017.3808

[36] Kim HJ , Eoh KJ , Kim LK , et al. The long noncoding RNA HOXA11 antisense induces tumor progression and stemness maintenance in cervical cancer. Oncotarget. 2016;7:83001‐83016. 27792998 10.18632/oncotarget.12863 PMC5347748

